# Circadian Rhythm: Potential Therapeutic Target for Atherosclerosis and Thrombosis

**DOI:** 10.3390/ijms22020676

**Published:** 2021-01-12

**Authors:** Andy W. C. Man, Huige Li, Ning Xia

**Affiliations:** Department of Pharmacology, Johannes Gutenberg University Medical Center, 55131 Mainz, Germany; wingcman@uni-mainz.de (A.W.C.M.); huigeli@uni-mainz.de (H.L.)

**Keywords:** clock genes, inflammation, oxidative stress, circadian disruption, cardiovascular diseases

## Abstract

Every organism has an intrinsic biological rhythm that orchestrates biological processes in adjusting to daily environmental changes. Circadian rhythms are maintained by networks of molecular clocks throughout the core and peripheral tissues, including immune cells, blood vessels, and perivascular adipose tissues. Recent findings have suggested strong correlations between the circadian clock and cardiovascular diseases. Desynchronization between the circadian rhythm and body metabolism contributes to the development of cardiovascular diseases including arteriosclerosis and thrombosis. Circadian rhythms are involved in controlling inflammatory processes and metabolisms, which can influence the pathology of arteriosclerosis and thrombosis. Circadian clock genes are critical in maintaining the robust relationship between diurnal variation and the cardiovascular system. The circadian machinery in the vascular system may be a novel therapeutic target for the prevention and treatment of cardiovascular diseases. The research on circadian rhythms in cardiovascular diseases is still progressing. In this review, we briefly summarize recent studies on circadian rhythms and cardiovascular homeostasis, focusing on the circadian control of inflammatory processes and metabolisms. Based on the recent findings, we discuss the potential target molecules for future therapeutic strategies against cardiovascular diseases by targeting the circadian clock.

## 1. Introduction

The behavioral patterns of human activities in modern society have changed dramatically in terms of day–night rhythms. Longitudinal studies have shown that shift workers are at higher risk for metabolic and cardiovascular complications [1,2]. Shift workers may have higher chances of getting adverse health outcomes via multifactorial pathways, including psycho-social factors, insomnia, reduced physical activity, altered nutrition quality, and reduced light exposure [3]. Night shift workers and individuals with sleep disorders exhibit exacerbated blood vessel stiffening and increased chance of getting coronary artery diseases [4,5,6,7]. Studies have reported that night shift workers have worse metabolic profiles and electrocardiographic changes than normal workers [6,8]. A “non-dipping” systolic blood pressure exhibits a night/day ratio of >0.9 [9], and is associated with various vascular and metabolic dysfunctions [10]. Chronically, shift workers may develop a “non-dipper” status which increases the risk for hypertension [11]. The increased risk for cardiovascular complications in shift workers has raised concerns about the misalignment between the body metabolism and the circadian rhythm [12].

The intrinsic mechanism that responds to the environmental light–dark cycle, is called circadian (derived from Latin “*circa diem*”, meaning “about a day”). The circadian clock has intimate relationships with many important physiologies and pathways. The intrinsic circadian clock has an approximately 24-h oscillation cycle that responses to abiotic/biotic factors and orchestrates biological processes in adjusting to daily environmental changes [13,14]. The circadian clock components include networks of genes and molecules in the core and peripheral tissues. The intrinsic circadian clock is self-sustaining, through the control of negative feedback loops of the molecular clock. Zeitgeber (German for time-giver) refers to the internal or external factors, which can cue the circadian clock and modulate the circadian rhythm from the molecular to the behavioral level [15,16].

The core circadian clock is located in the hypothalamic suprachiasmatic nucleus (SCN) [17]. The SCN is the master clock that synchronizes neurons and coordinates circadian outputs. The circadian outputs are triggered by the photic information transmitted from the retina [18]. Aberrant light exposure disturbs the function of SCN and causes circadian disruption [19]. The central clock in the SCN regulates peripheral clocks and coordinates circadian gene expression. This process can be regulated directly by neuronal and hormonal signaling. It can also be regulated by driving appetite, blood pressure and body temperature indirectly [20,21].

Although SCN is the master clock regulator, peripheral tissues are capable of local and autonomous clock regulation [22]. Peripheral clocks are found in almost all the peripheral tissues including immune cells, adipose tissues, kidney, liver and also the tissues of the vascular system [17]. The circadian gene expression and function in these peripheral tissues can be affected by the peripheral clocks [23]. In mice, 6% and 4% transcripts of protein-coding genes show circadian oscillations in the heart and aorta, respectively [24]. Ex vivo study has revealed that around 8% of macrophage transcriptomes are under local circadian regulation. The macrophages are isolated from spleen, lymph node, and peritoneum in this study [25]. These genes include those related to the molecular clock, glucose and lipid metabolism, and vascular integrity. These results suggest the importance of the peripheral clock regulation.

Atherosclerosis results from the progressive accumulation of lipids and fibrous elements in large arteries. Atherosclerosis is the primary cause of cardiovascular diseases, stroke and myocardial infarction [26]. Obesity, diabetes mellitus, dyslipidemia, hypertension, and smoking are well-known risk factors for atherosclerosis and other cardiovascular diseases. Accumulating evidence suggests, that the circadian disruption is also a critical factor leading to atherosclerosis [27,28,29].

Physiological parameters of the cardiovascular system, such as blood pressure, heart rate, and endothelial function, exhibit diurnal variations within a day [30,31,32,33,34]. A normal day–night difference in blood pressure is essential in maintaining cardiovascular health. Clinical studies have suggested the attribution of diurnal variations of cardiovascular parameters in the pathophysiology and pathogenesis of cardiovascular complications [30,31,32,33,34]. In humans, frequencies of thromboembolic and cardiovascular events exhibit clear diurnal variations, which peak during the morning-to-noon period [35,36]. Plasma levels of lipids display circadian oscillations independent of food intake [37]. It suggests that the intrinsic biological clock is an important regulator of the body lipid metabolism. Levels of immune cells and pro-inflammatory cytokines show circadian fluctuations [38], while the activity of the immune system is strongly linked to the circadian rhythm [39,40]. Therefore, the desynchronization of the clock and the misalignment between the circadian rhythm, lipid metabolism, and immune system could result in the development of dyslipidemia and inflammation and contribute to the risk of atherosclerosis. This suggests that the circadian rhythm could be a novel therapeutic target for and cardiovascular diseases, especially arteriosclerosis and thrombosis. However, the underlying mechanisms remain elusive.

In this review, we summarize the recent findings on the role of the circadian rhythm in the progression of arteriosclerosis and thrombosis. The possible treatment of atherosclerosis and thrombosis through targeting circadian clocks is discussed.

## 2. Circadian Rhythm and Arteriosclerosis

The blood clotting is a protective mechanism against bleeding events. However, the formation of blood clots in vasculatures can also lead to severe cardiovascular events including ischemic stroke, myocardial infarction, and sudden cardiac arrest. Atherosclerosis is a chronic inflammatory condition that is initiated by endothelial dysfunction and the upregulation of adhesion molecules [41]. These promote the recruitment of leukocytes to the inflamed endothelium and the formation of atherosclerotic plaques. Atherosclerosis is the underlying pathology of most cardiovascular diseases. Thrombosis can be caused by atherosclerosis and occlude the blood vessel. Thrombolysis can break down clots to maintain normal blood flow in the blood vessels [42]. The balance between clotting and thrombolysis is regulated in a circadian manner [43].

The ability of the vascular endothelium to cause vasodilation is important in protecting against cardiovascular disease. The vascular endothelial nitric oxide synthase (eNOS) has anti-atherosclerotic functions. NO produced by eNOS can inhibit platelet aggregation and regulate the patency of vessels [44,45]. Dysfunction of eNOS causes NO imbalance in the endothelium and leads to endothelial dysfunction [46]. There is evidence showing that the peripheral circadian clock can regulate eNOS expression, which in turn, modulates the diurnal variation of blood pressure [47,48,49]. Normally, endothelium-dependent vasorelaxation is reduced during the light cycle, due to the lowered NO production in the morning [50]. The deterioration of NO production might contribute to the morning peak of incidence of cardiovascular diseases [44,45,51].

Monocytes and macrophages are the key players in inflammatory response and atherogenesis [51]. When the blood cholesterol level is high, inflammatory Ly6c^hi^ monocytes adhere to the inflamed endothelium and differentiate into lesion macrophages [51]. This is the critical step for the initiation and exacerbation of atherosclerotic plaque formation. The functions of the macrophages in atherosclerotic lesions, such as proliferation, M1, and M2 polarization, apoptosis and cholesterol efflux are important for the progression of atherosclerosis [52]. In addition, the numbers of hematopoietic cells and the production of cytokines have been shown to oscillate in diurnal rhythm and are orchestrated by the molecular clock [40,53].

Recent study reveals that circulating leukocyte counts peak during the inactive phase in the murine blood [54]. By contrast, leukocyte counts in other tissues, such as bone marrow, skeletal muscle, and the heart, peak during of the active phase [54,55]. The detailed mechanism of the leukocyte counts oscillation is not well-known, it is likely regulated by networks of chemokines and endothelial adhesion molecules [54].

Expression of many hemostasis-related molecules has been shown to align with the circadian rhythm. The fibrinolytic activity is lowest at night and starts to increase before morning. This circadian rhythm has first been described since the 1950s [56]. In human, the number of platelets peaks in the afternoon and is most active in the morning [57,58]. Markers of platelet activation, including β-thromboglobulin (β-TG) and platelet factor 4 (CXCL4), are expressed in a circadian rhythm and peak in the afternoon [59]. Activities of coagulation factors, including factor VII, factor VIII, factor IX, and von Willebrand factor (vWF) oscillate in circadian rhythms [57,59,60,61]. Factor X activity (Xa) and D-dimers, the markers of fibrinolysis, peak in the morning [61].

There is a correlation between the circadian oscillations of interleukin-6 (IL-6) and fibrinogen. The IL-6 level peaks in the early hours of the night, while the fibrinogen level peaks later in the morning [61,62].

The activities of plasminogen activator inhibitor-1 (PAI-1) and tissue plasminogen activator (t-PA) oscillate in phase opposition. The activity of PAI-1 peaks in the morning, while the activity of t-PA peaks in the afternoon [63,64]. Protein C, protein S, and antithrombin (AT) are also expressed in a circadian rhythm [65]. The expression of thrombomodulin is controlled by the peripheral clock in endothelial cells [66]. Matrix metalloproteinase (MMP-1 and MMP-3), collagen IIIA1, transgelin, and calponin are involved in the stability of atherosclerotic plaques. In mouse smooth muscle cells, these molecules have been shown to express in a circadian pattern [67].

**Figure 1:**
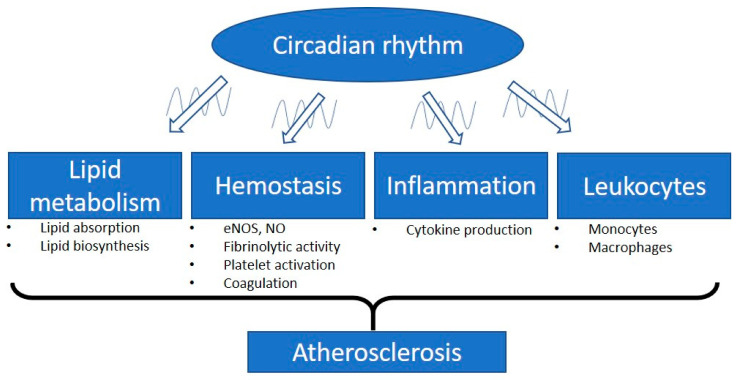
Involvement of the circadian rhythm in atherosclerosis. The intrinsic biological clock has an oscillation cycle of approximately 24 h. The circadian clock controls different physiological parameters of the cardiovascular system such as blood pressure, heart rate, and endothelial function. Circadian disruption is a critical factor leading to atherosclerosis and cardiovascular diseases. Plasma lipid levels are mediated by the balance of lipid absorption and biosynthesis. The lipid plasma levels display circadian oscillations and are independent of food intake. Vascular functions, especially the related endothelial nitric oxide synthase (eNOS) expression and nitric oxide (NO) production, are regulated by the peripheral circadian clock. Many of the important molecules involved in hemostasis have been shown to align with circadian rhythms, including molecules responsible for fibrinolytic activity, platelet activation, and coagulation. Dysregulation of the circadian rhythm leads to inflammation. Proinflammatory cytokines are expressed in a circadian manner. In response to inflammatory stimuli, circulating counts of leukocytes and the function of monocytes/macrophages are modulated by the circadian clock. Therefore, the misalignment of the circadian clock with these parameters could lead to the progression of atherosclerosis.

These findings suggest the significant involvement of the circadian clock in hemostasis. The misalignment between the circadian and the fibrinolytic system may increase the risk of cardiovascular events [68] (Figure 1).

## 3. Circadian Disruption and Vascular Complications

The intrinsic circadian clock worsen during aging [69] and obesity [70]. The amplitudes of circadian rhythms are dampened, or the peaks of the rhythms are shifted in the worsened clocks [71]. Recent evidence indicates that the circadian rhythm in the vasculature is important for vascular function and health [72,73,74]. The misalignment between the clock and metabolism can cause cardiovascular complications, including pathological vascular remodeling, vascular senescence, hypertension, stenotic atherosclerotic lesions, vascular graft failure, and diabetic vasculopathies [44,75,76,77,78]. Compromised circadian clock machineries in the vasculature, including mutations or polymorphisms in clock genes and the reduction in the oscillation amplitude of the clock genes, are observed in models of obesity and cellular senescence [70,79].

Proinflammatory stimuli can disrupt circadian rhythms and suppress the oscillation amplitude of clock components with negative feedback in isolated macrophages, whereas anti-inflammatory signals can improve circadian rhythms [80,81]. In mice, long-term sleep fragmentation increases circulating levels of inflammatory cytokines, including IL-1β, IL-6, and TNF-α [82]. Long-term sleep fragmentation can reduce the phosphorylation and transcriptional activity of cyclic AMP response element-binding protein (CREB) [82], whereas the downregulation of CREB may contribute to the pathological responses to vascular injury and plaque progression [83].

Apolipoprotein E *(ApoE)^−/−^* mouse is a widely used murine model for atherosclerosis [84]. *ApoE*^−/−^ mice fed with Western diet for four weeks have accelerated atherosclerosis and exhibit an altered circadian expression profile of cardiac clock genes and apoptosis-related genes (*c-Myc* and *p53*) [85]. This suggests an interrelationship between the circadian clock and lipid metabolism. Severe disruption of circadian rhythms by exposing to constant light exacerbates atherosclerosis in male, but not in female *ApoE^−/−^* mice. When the circadian rhythm is disrupted, male *ApoE*^−/−^ mice have increased serum LDL level [86]. In hyperlipidemic female APOE*3-Leiden.CETP mice, exposure to 12-h shift of light-dark cycles for 15 weeks causes a significant increase in atherosclerosis, while male mice do not. Higher lesion macrophage contents, increased inflammation and oxidative stress are observed in these hyperlipidemic mice. These suggest the involvement of the immune system in disrupted circadian-induced atherosclerosis development [87]. The underlying mechanisms of the observed gender difference are not yet studied, but it is hypothesized that these could be due to the differences in circulating sex hormones.

Interestingly, time of surgery can affect the thrombus formation and resolution response in mice. In a recent study on thrombus formation, mice ligated at 12:00 p.m. have lower survival rate than the mice ligated at 7:00 a.m. [88]. The thrombi sizes of the 12:00 p.m.-ligated mice are larger than that of the 7:00 a.m.-ligated mice, while the gene expression of MMP9 is significantly reduced in the 12:00 p.m.-ligated mice. These suggest that treatment time may affect the response of mice to thrombosis [88].

## 4. Clock Components

The intrinsic molecular clock consists of interlocked transcription–translation feedback loops of clock genes and proteins [89]. The master clock regulators include brain and muscle aryl hydrocarbon receptor nuclear translocator-like protein 1 (BMAL1 or ARNTL), circadian locomotor output cycles kaput (CLOCK), Period 1/2/3 (PER1/2/3), and Cryptochrome 1/2 (CRY1/2). BMAL and CLOCK are important transcription factors. PER1/2/3 and CRY1/2 are transcriptional modulators [22,89]. In mouse aorta, PER1/2 and CRY1/2 mRNA levels peak at early night cycle and trough at day cycle [90], while the protein expression of BMAL1 peaks at the beginning of the day cycle and troughs at the night cycle. Similar phase difference relationships of the clock genes are reported in both human and mouse smooth muscle cell model in vitro [90].

During the light phase, BMAL1 can dimerize with CLOCK and bind to the E-box regulatory sites (5′-CACGTG-3′) in the promoter regions. This binding can activate the transcription of many circadian proteins including PER1/2/3 and CRY1/2 [17]. When PERs and CRYs proteins accumulate in the cytoplasm and reach certain levels, they can dimerize to form repressor complex and translocate into the nucleus, where they inhibit the CLOCK:BMAL1-mediated transcription, forming a negative feedback loop [17,91].

The reinforcing loops of the molecular clock are composed by the circadian nuclear receptors, reverse ERB (REV-ERB α/β) and retinoic acid receptor-related orphan receptors (RORα/β/γ). These loops are important in controlling the rhythmic gene transcriptions of BMAL1 and CLOCK. Both REV-ERB and ROR interact with the ROR response elements at the promotor regions of BMAL1 and CLOCK. REV-ERBα negatively regulates the gene expression of BMAL1 and CLOCK [92], while RORα and RORγ positively regulate the gene expression of BMAL1 and CLOCK [93,94].

**Figure 2:**
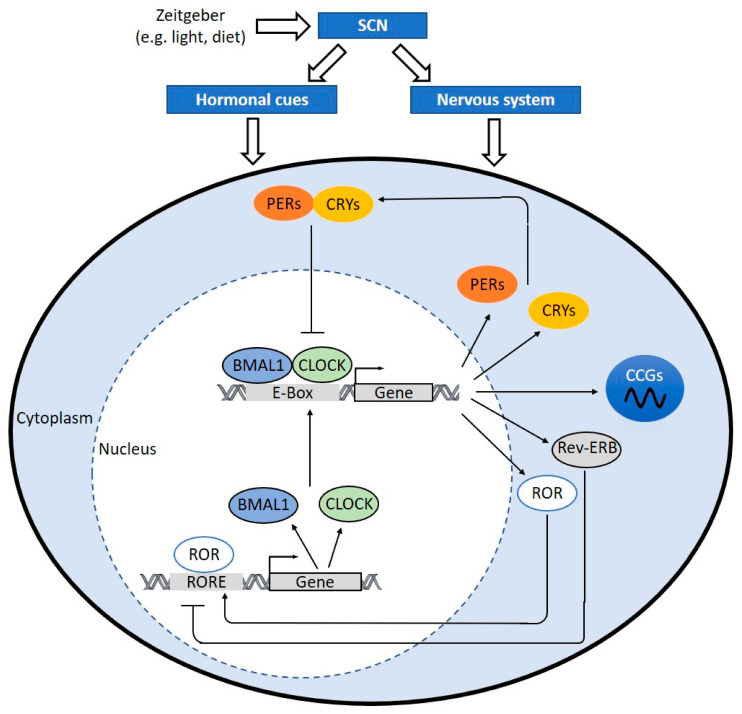
The molecular clock mechanism. When the hypothalamic suprachiasmatic nucleus (SCN) is triggered, the SCN translates signals into hormonal cues and nerve impulses, which can regulate the peripheral clock. In cells, the heterodimer of circadian locomotor output cycles kaput (CLOCK) and brain and muscle aryl hydrocarbon receptor nuclear translocator-like protein 1 (BMAL1) binds to E-box sequence (5′-CACGTG-3′) in the promoter and activates the transcription of Period (PER)1/2/3, Cryptochrome (CRY)1/2, retinoic acid receptor-related orphan receptors (ROR), and reverse ERB (Rev-ERB). Dimerized PERs and CRYs translocate into nucleus and interfere CLOCK:BMAL1-mediated transcription. REV-ERB negatively regulates BMAL1 and CLOCK expression. RORs positively regulate BMAL and CLOCK expression via ROR response elements (RORE) at their promotor regions. The clock drives rhythmic expression of clock-controlled genes (CCGs) through CLOCK:BMAL1-mediated activation of circadian promoter elements, including E-boxes, D-boxes, and ROR response elements. CCGs encode important proteins involved in processes of atherosclerosis development, hemostasis, inflammation, lipid metabolism, and macrophage trafficking.

The molecular circadian clock can modulate the rhythmic expression of clock-controlled genes (CCGs) by activating different circadian promoter elements. These regulatory promoter elements include D-boxes, E-boxes, and ROR response elements [93]. CCGs encode different important proteins involved in cellular metabolisms and inflammatory responses [95,96] (Figure 2). Although identical clock machineries are found in most cells, the circadian expression pattern of CCGs are highly tissue-specific or even cell-type-specific [14]. Post-translational modifications also contribute to the regulation of circadian clock gene expression [14,97].

## 5. Clock Components and Vascular Complications

### 5.1. BMAL1 and CLOCK

*Bmal1*-deficient mice exhibit reduced lifespans and premature aging with related pathologies. Sarcopenia, loss of visceral and subcutaneous adipose tissues, osteoporosis, organ shrinkage, changes in blood cell composition, and loss of pressor response to stress are observed [98]. Young *Bmal1*-deficient mice have increased pathological remodeling and vascular injuries with reduced blood flow. Arteries from *Bmal1*-deficient mice loss the ability to narrow (inward remodeling), which is reminiscent of the similar response in *eNOS* knockout mice. Arteries from *Bmal1*-deficient mice also have significant increase in collagen deposition in the medial layer, which leads to wall thickening [78].

Transplantation of arteries from *Bmal1*-knockout mice to wild type mice leads to the development of atherosclerosis in the transplanted blood vessels without affecting the systemic hemodynamics. This suggests a critical role for autonomous peripheral circadian clocks [99]. Most downstream target genes of BMAL1 appear to be tissue specific and play differential pathophysiological roles in atherosclerosis [100,101]. BMAL1 and CLOCK can directly regulate the expression of the prothrombotic mediator von Willebrand factor (vWF). The expression levels of vWF, fibrinogen, and plasminogen activation inhibitor-1 (PAI-1) are increased in *Bmal1*^−/−^ mice, which lead to accelerated arterial thrombus formation [102].

BMAL1 can regulate the expression of inflammatory marker CCL2. CCL2-CCR2 chemokine axis is involved in the early lesion development in mice [95]. The circadian expression of monocyte chemoattractant protein-1 (MCP-1) in macrophages is regulated by BMAL1-mediated activation of nuclear factor kappa-light-chain-enhancer of activated B cells (NF-κB) [103]. In *Bmal1*^−/−^ mice, both basal and tumor necrosis factor-α (TNF-α)-induced NF-κB activations are upregulated in macrophages [104,105]. BMAL1 is required to maintain the diurnal oscillation of inflammatory Ly6c^hi^ monocytes and their trafficking to sites of acute inflammation [95].

Mice with monocytes- and macrophages-specific *Bmal1*-deficiency have enhanced atherosclerosis in carotid arteries. These mice also have increased total number of macrophages and Ly6c^hi^ infiltrating monocyte-macrophages in atherosclerotic lesions. These suggest the importance of BMAL1 in maintaining normal macrophage functions [101]. Endothelial-specific *Bmal1*^−/−^ mice maintain the circadian rhythm of blood pressure, but their blood pressure in the active phase is lower than the blood pressure of control mice [106]. Specific deletions of *Bmal1* in endothelial and hematopoietic cells result in accentuated vascular injuries [107]. Smooth muscle-specific *Bmal1*^−/−^ mice have reduced amplitude of blood pressure oscillation without affecting locomotor activity. These suggest that the vascular BMAL1 can regulate blood pressure master clock independently [108]. However, further studies on the atherosclerotic development on these mice are needed to dissect the role of BMAL1 in peripheral tissues.

*Clock*-deficient mice have a significantly reduced lifespan, which is about 15% shorter than that of wild type littermates [109]. *Clock* mutant mice show phenotypes that are reminiscent of accelerated aging [110], obesity, and hypertension [111,112]. Macrophages isolated from *Clock* mutant mice have higher intracellular levels of total, free, and esterified cholesterol. The macrophages from these mice also have reduced expression of the ATP-binding cassette transporter (ABCA1 and ABCG1) and blunted abilities to efflux cholesterol to ApoA1 [113]. Both *Bmal1^−/−^* and *Clock^mut^* animals loss the circadian variation in glucose and triglycerides [111]. Either deletion or knockdown of *Clock* or *Bmal1* abolishes the rhythmic oscillation of genes involved in lipid metabolism in the liver, including acetyl co-A carboxylase (ACC), acetyl-CoA synthetase (ACS), and sterol regulatory element-binding protein-1c (SREBP-1c). These suggest important roles of CLOCK and BMAL in modulating glucose homeostasis and lipid profiles in vivo [114,115].

### 5.2. CRY1/2

Atherosclerotic patients have lower serum CRY1 mRNA level [116]. In mice, deletion of *Cry1* and *Cry2* leads to constant elevation of proinflammatory cytokines including IL-6, TNF-α and inducible nitric oxide synthase (iNOS) [117]. CRY1 and CRY2 have been shown to interact with the glucocorticoid receptor in a ligand-dependent fashion [118]. Both *Cry1*- and *Cry2*-deficient mice exhibit glucose intolerance and have elevated plasma glucose levels in response to acute feeding after a 12 h overnight fasting [118]. In *ApoE*^−/−^ mice, overexpression of CRY1 by adenovirus-mediated gene transfer significantly reduces the expression of proinflammatory markers, including IL-1 and 6, TNF-α, NF-κB, and macrophage inflammatory protein-1α (MIP-1α). These mice also have reduced plasma total cholesterol (TC), triglyceride (TG), and low-density lipoprotein cholesterol (LDL-C) levels. The mice are protected against plaque development [116].

### 5.3. PER1/2

Deficiency of *Per1* and *Per2* in mice results in altered circadian rhythms [119,120]. Although *Per1* and *Per2* mutant mice cannot be distinguished morphologically from wild type mice at birth, phenotypes of premature aging are observed from the age of 12 months, including faster decline in fertility and loss of soft tissues [121]. *Per2* mutant mice have impaired clock resetting ability and lose circadian rhythms in constant darkness [122].

PER2 is a major regulator of lipid metabolism by controlling the proadipogenic activity of peroxisome proliferating activated receptor (PPARγ) [123]. Both PER1/2-null mice and PER2-null mice have lower hepatic TG levels [124].

The transplantation of arteries from *Per1/2^−/−^* mice to wild type mice leads to the development of atherosclerosis in the transplanted graft, which suggests the important role of peripheral PER1/2 in the vascular system [99].

During the transition from resting to active phase, endothelium-dependent relaxation response is increased in the aortae of wild-type mice, but not in PER2 mutant mice, confirming a circadian control of endothelial function [125]. PER2 mutant mice also show increased vascular senescence and endothelial dysfunctions [126]. The blood vessels from *Per2* mutant mice have reduced production of endothelial-derived relaxation factors (EDRF), including prostaglandins and nitric oxide (NO). The expression of vasoconstrictor cyclooxygenase-1 (COX1) is increased in *Per2* mutant mice. The aortae from *Per2* mutant mice show a significant reduction of NO dependent endothelial function and enhanced lesion development [126]. Angiogenic response to hind limb ischemia is blunted in *Per2* mutant mice [125]. *Per2* mutant mice show diabetes-like vascular phenotypes such as retinal vascular damage and neuronal loss [127].

### 5.4. REV-ERB

*Rev-erbα*-mutant mice have increased adiposity and mild hyperglycemia without insulin resistance after high-fat diet (HFD) [128]. Liver-specific *Rev-erbα*-knockout mice have increased serum levels of cholesterol, TGs, and free fatty acids [129]. REV-ERBα modulates the infiltration of inflammatory macrophages by inhibiting the expression of *Ccl2* [130]. Knocking-down of *Rev-erbα* in hematopoietic cells enhances atherosclerotic lesion formation in mouse aorta and increases the inflammatory phenotype of macrophages both in vitro and in vivo [131]. Pharmacological activation of REV-ERBα reduces atherosclerotic lesion formation and promotes anti-inflammatory M2 markers expression [131].

A synthetic REV-ERB agonist, SR9009, has been shown to activate REV-ERB activity and leads to reduced size of atherosclerotic plaque in atherosclerosis-prone LDL receptor (*Ldlr*)-deficient mice [132]. SR9009 administration can normalize cardiac gene expression and function by mediating the circadian clock-controlled processes in the heart [133]. REV-ERB agonist treatment can reduce the polarization of bone marrow-derived mouse macrophages (BMDMs) to proinflammatory M1 macrophages and increase the polarization of BMDMs to anti-inflammatory M2 macrophages [132]. These indicate the possibility of targeting REV-ERBs for the treatment of atherosclerosis. However, the outcome of SR9009 treatments may not be solely attributed to its effect on the circadian rhythms. A recent study reported REV-ERB-independent effects of SR9009 on cell proliferation and metabolism [134].

In short summary, disruption of the circadian clock at different nodes (BMAL1, CLOCK, CRYs, PERs, and REV-ERB) promotes atherosclerosis. These clock components are involved in the homeostasis of glucose and lipid metabolism by controlling the circadian expression and activities of key regulatory enzymes. Maintaining the high amplitude oscillation of these clock components may be a potential strategy for prevention against atherosclerosis and other cardiovascular complications.

## 6. Targeting the Circadian Clock for the Treatment of Atherosclerosis

### 6.1. The Role of SIRT1 in Regulating the Circadian Rhythm

SIRT1 is a nicotinamide adenine dinucleotide (NAD+)-dependent protein deacetylase. SIRT1 is well-known for its vascular protective effects, including enhancing endothelium-dependent vasodilatation, promoting endothelial angiogenesis and migration, suppressing vascular inflammation, preventing endothelial senescence and adverse arterial remodeling, and suppressing foam cell formation. These effects of SIRT1 made it an important player in protecting against atherosclerosis [135]. SIRT1 can suppress vascular inflammation by regulating NF-κB activity through deacetylating K310 on the p65 subunit [136]. Reduced SIRT1 level has been shown to upregulate NF-κB and increase inflammatory responses in monocytes/macrophages, myeloid cells, and endothelial cells [137,138,139,140]. SIRT1 can regulate the expression of liver X receptors (LXRs), which confers beneficial effects in lipid metabolism and suppresses foam cell formation [141].

SIRT1 is highly involved in the crosstalk between the circadian clock and energy metabolism [142]. SIRT1 is required for the high-magnitude circadian transcription of circadian clock genes including *Clock, Bmal1, Cry*s, and *Per*s [143]. *Sirt1*-deficienct mice have disrupted circadian rhythms and altered amplitudes of *Per1/2* and *Cry1/2* expression [144]. SIRT1 directly activates the transcription of *Bmal1*, and increases the oscillating amplitude of other clock genes via peroxisome proliferator-activated receptor gamma coactivator 1-alpha (PGC-1α) [145]. SIRT1 can directly deacetylate BMAL1 and PER2 to affect their transcriptional activities [146]. The deacetylation of PER2 by SIRT1 can lead to PER2 degradation [143]. A negative reciprocal relationship exists between SIRT1 and PER2 [144]. PER2 negatively regulates *Sirt1* transcription through competing CLOCK/BMAL1 binding sites at SIRT1 promotor [144].

One of the transcriptional targets of the CLOCK:BMAL1 dimer is nicotinamide phosphoribosyltransferase (NAMPT), which is an enzyme required for the biosynthesis of NAD+ [147,148]. The circadian clock can exert a rhythmic regulation on SIRT1 activity via NAMPT [147,148]. While rhythmic NAD+ level affects SIRT1 activity, SIRT1 may in turn affect the circadian levels of metabolites including NAD+ and acetyl-CoA. The intracellular acetyl-CoA level is controlled by SIRT1-mediated deacetylation of acetyl-coenzyme A synthetase 1 (ACS1) [149]. SIRT1 can be expressed in a circadian manner. The expression of SIRT1 is in high oscillating rhythms in young animals, whereas the rhythmic oscillations of SIRT1 expressions are nearly flattened in aged animals. The components of this amplifying loop, including SIRT1, PGC-1α, and NAMPT, are critical in the intrinsic circadian regulation [145].

In *ApoE*^−/−^ mice, abnormal exposure to light exacerbates atherosclerotic plaque formation and circadian disruption, which are associated with altered expression of clock genes, lipid metabolism genes and SIRT1 [150].

Collectively, SIRT1 may serve as an important link between the circadian clock and lipid-related gene oscillation. SIRT1 has many beneficial effects in protection against atherosclerosis. These findings raise the potential use of SIRT1 activators in modulating the circadian rhythm and preventing atherosclerosis.

### 6.2. Krüppel-Like Factors (KLFs), Circadian Rhythms and Atherosclerosis

Krüppel-like factors (KLFs) belong to an evolutionarily conserved zinc-finger transcription factors family, which bind to CACCC elements and GC-rich regions of DNA. Members of the KLF family are key regulators of important biological processes, including cell differentiation, proliferation, apoptosis, metabolism, and anti-polymicrobial activity [151,152]. The transcriptions of the KLFs are regulated by direct promoter binding of CLOCK and BMAL1 [153,154].

In endothelial cells, overexpression of KLF2 leads to the secretion of vascular protective miRs-143/145 in microvesicles [155]. miRs-143/145 can reduce atherosclerosis by targeting critical genes for vascular smooth muscle cells dedifferentiation, including *Mmp3,* ETS like-1 protein *(Elk1)* and calcium/calmodulin-dependent protein kinase type II delta chain *(Camk2d)* [155]. KLF2 represses endothelial inflammation and modulates anti-thrombotic transcription. KLF2 directly binds to the promoter of thombomodulin-1 and increases the expression of this potent anti-thrombotic and anti-inflammatory factor [156]. KLF2 inhibits thrombin-mediated endothelial activation by preventing the transcription of protease-activated receptor (PAR-1). PAR-1 is a thrombin receptor [157]. In vivo, *Klf2*^+/−^*ApoE^−/−^* mice are more susceptible to the development of atherosclerotic lesion compared with *Klf2*^+/+^*ApoE^−/−^* mice [158]. Post-natal deletion of KLF2 leads to a thrombotic phenotype in mice, while overexpression of KLF2 protects mice from thrombus formation. Overexpression of KLF2 decreases the expression of thrombotic genes coding for iNOS and MCP-1 in peritoneal macrophages. The expression levels of PAR-1 and thrombomodulin in endothelial cells are also reduced by the KLF2 overexpression [159]. Myeloid-specific KLF2 deletion in mice with the *ApoE*^−/−^ background promotes vascular oxidative stress and atherosclerosis [160]. In human, monocytes from atherosclerotic patients have reduced *Klf2* expression [161].

KLF4 regulates the reverse cholesterol transport out of the vascular wall and inhibits the inflammation by inducing the expression of cholesterol-25-hydroxylase (Ch25h) and LXR in endothelial cells [162]. Overexpression of KLF4 in endothelial cell protects against the pathogenesis of atherosclerosis and thrombosis [163]. Loss of myeloid KLF4 promotes atherosclerosis, whereas macrophages specific *Klf4*-deficient mice have increased inflammation in response to oxidized phospholipids [164].

KLF10 is a regulator of bone physiology. KLF10 also regulates glucose and lipid metabolism in liver [165]. 36% and 23.4% of KLF10- regulated genes are involved in lipid and carbohydrate metabolisms respectively [165]. *Klf10^−^*^/−^ male mice had 20% higher blood glucose levels than wild-type mice, while *Klf10^−^*^/−^ female mice exhibit a 20% increase of plasma TG level compared to wild-type mice [166].

In *ApoE*^−/−^ mice, *Klf14* expression is increased in the aorta compared to wild-type mice [167]. Overexpression of KLF14 in macrophages increases the production of inflammatory cytokine, TC and cholesteryl ester content, reminiscent of the phenotype of atherogenic foam cells [168].

Both rat aortic vascular smoother muscle cells exposed to oxidized phospholipids and human atherosclerotic tissues have markedly reduced KLF15 expression [169]. Both systemic and smooth muscle-specific *Klf15*-deficient mice exhibit an aggressive inflammatory vasculopathy in diet-induced atherosclerosis [169]. Recently, KLF15 has been shown to regulate circadian susceptibility to ischemia reperfusion injury in the heart, while KLF15 expression is reduced in the heart of patients with cardiomyopathies [170]. These suggest that KLF15 may play an important role in atherosclerosis.

Collectively, circadian oscillation of KLFs contributes to the rhythmic regulation of their target genes. Dysregulation of KLFs may promote atherosclerosis. To clarify the pharmacological potential for arteriosclerosis treatment with KLFs as target, further studies of the detailed association between KLFs, circadian clock, and atherosclerosis are needed.

### 6.3. Polyphenols and the Circadian Clock

Polyphenols are secondary metabolites of plants, which have been widely studied on their beneficial effects as antioxidants [171,172]. Polyphenols can affect cholesterol metabolism via bile acid biosynthesis [173]. In addition to their antioxidative and anti-inflammatory properties, polyphenols may prevent atherosclerosis by modulating the circadian clock. Polyphenols interact with circadian clock by modulating the amplitude and period of the clock gene oscillations [174,175,176]. The use of polyphenols in entraining the circadian clock has been widely studied and reviewed [171,177,178,179].

Resveratrol is a well-known activator of SIRT1 [180]. Resveratrol has been shown to attenuate HFD-induced obesity in mice by normalizing the nearly flattened circadian expression of PER2, CLOCK, and BMAL1 [181]. In *ApoE*^−/−^ mice, resveratrol can inhibit either TMAO-induced atherosclerosis or HFD- and lipopolysaccharides (LPS)-induced atherosclerosis [182].

Equol, a soy bean-based isoflavone-derived metabolite, can significantly reduce the atherosclerotic lesions, serum TG, TC and LDL-cholesterol levels, and increase HDL-cholesterol level in *ApoE*^−/−^ mice fed with HFD [183].

**Figure 3:**
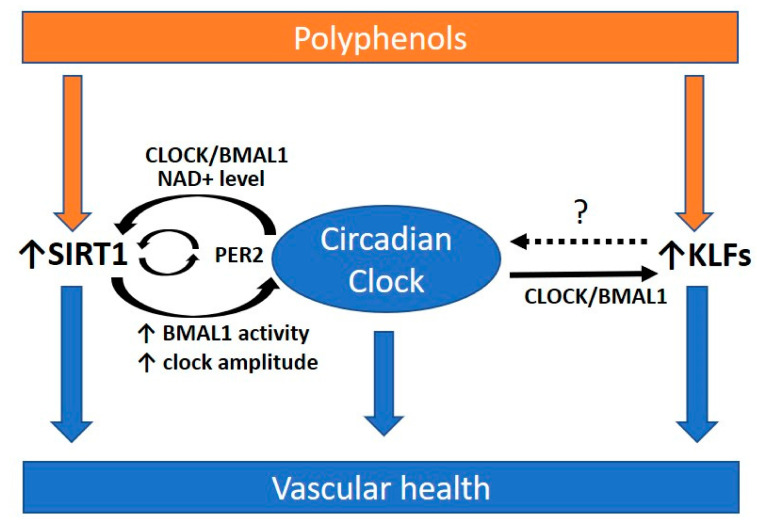
Polyphenols and the circadian rhythm for promoting vascular health. Polyphenols are secondary metabolites of plants, which have been widely studied for their beneficial effects on cardiovascular health. Polyphenols may be beneficial in preventing atherosclerosis via modulating the circadian clock. Polyphenols have been shown to enhance the expression of Krüppel-like factors (KLFs) and Sirtuin 1 (SIRT1), which are highly associated with the circadian clock. SIRT1 and KLFs are important players in protecting against atherosclerosis. SIRT1 interacts with the circadian clock and is required for high-magnitude circadian transcription of circadian clock genes. The circadian clock can regulate SIRT1 activity via the oscillating NAD+ level. The transcription of KLFs is regulated by direct promoter binding of CLOCK:BMAL1. However, whether KLFs have a feedback control to the circadian clock remains unclear (dotted arrow and ?). A functional circadian clock is required for preventing atherosclerosis. PER2, period 2. BMAL1, brain and muscle aryl hydrocarbon receptor nuclear translocator-like protein 1; CLOCK, circadian locomotor output cycles kaput. NAD+, oxidized nicotinamide adenine dinucleotide.

Polyphenols have been shown to enhance the expression of KLFs [184,185,186]. These suggest the beneficial effect of polyphenols against circadian disruption is possibly acting through SIRT1 and KLFs (Figure 3). Since most studies about polyphenols focused on their antioxidant and anti-inflammatory properties, future study direction may include their effects on modulating circadian rhythms and atherosclerosis treatment.

## 7. Summary and Future Directions

In summary, a large body of evidence suggests that the intrinsic circadian clock plays an important role in atherosclerosis in many aspects. The combinations of the central clock and peripheral clocks in the blood vessels, leukocytes and monocytes/macrophages orchestrate the normal hemodynamics and inflammatory responses. When the circadian rhythm is disrupted, the sequential inflammatory processes, endothelial dysfunction and lipid imbalance could promote the development of atherosclerotic lesions.

However, most of the study results are based on global clock gene knockout mice rather than tissue-specific knockout mice. Although SCN is the master clock, peripheral tissues are able to regulate the clock locally and autonomously, and the peripheral clocks are also important for regulating the function of local tissues [22]. Local homeostatic signaling pathways can affect circadian genes expression and function in the peripheral tissues [23]. We have mentioned a few reports demonstrated that the implantation of circadian disrupted tissues can cause atherosclerosis in normal mice. These results demonstrate and highlight the importance of peripheral clocks. The effects of the core clock and peripheral clocks must be clearly discriminated in studying their effects in cardiovascular diseases. Therefore, it can be more informative for in vivo studies to use cell type (i.e., endothelium, macrophages, or smooth muscle cells)-specific knockout mice in the future.

Ex vivo experiments have reported varied functions depending on the tissue collecting time in mouse heart and aorta, which suggested detailed records on the time of experiments should be included in future circadian studies [187].

SIRT1 and KLFs are important players in protecting against atherosclerosis. SIRT1 and KLFs are involved in the circadian regulation of cellular metabolisms. We propose the use of polyphenols as the potential supplement targeting SIRT1 and KLFs. It would be interesting to research other potential substances that regulate and link the circadian clock and atherosclerosis.

Based on current evidence, the circadian clock and its influence on cardiovascular diseases should be considered in the future studies for looking for therapeutic strategies of atherosclerosis and thrombosis. The pharmacokinetics of anti-atherosclerosis drugs may also be influenced by the circadian rhythm. Therefore, further studies on circadian rhythms could be needed to improve the effectiveness of medicines. Novel therapeutic targets entraining circadian clocks should be fully investigated.
